# Forecasting Air Temperature on Edge Devices with Embedded AI †

**DOI:** 10.3390/s21123973

**Published:** 2021-06-09

**Authors:** Gaia Codeluppi, Luca Davoli, Gianluigi Ferrari

**Affiliations:** Internet of Things (IoT) Lab, Department of Engineering and Architecture, University of Parma, Parco Area delle Scienze, 181/A, 43124 Parma, Italy; gaia.codeluppi@unipr.it (G.C.); luca.davoli@unipr.it (L.D.)

**Keywords:** internet of things, smart farming, EdgeAI, neural networks, greenhouse management, wireless sensor network, WSN, RNN, LSTM, ANN

## Abstract

With the advent of the Smart Agriculture, the joint utilization of Internet of Things (IoT) and Machine Learning (ML) holds the promise to significantly improve agricultural production and sustainability. In this paper, the design of a Neural Network (NN)-based prediction model of a greenhouse’s internal air temperature, to be deployed and run on an edge device with constrained capabilities, is investigated. The model relies on a time series-oriented approach, taking as input variables the past and present values of the air temperature to forecast the future ones. In detail, we evaluate three different NN architecture types—namely, Long Short-Term Memory (LSTM) networks, Recurrent NNs (RNNs) and Artificial NNs (ANNs)—with various values of the sliding window associated with input data. Experimental results show that the three best-performing models have a Root Mean Squared Error (RMSE) value in the range 0.289÷0.402 ${}^{\circ }C$, a Mean Absolute Percentage Error (MAPE) in the range of 0.87÷1.04%, and a coefficient of determination (R${}^{2}$) not smaller than 0.997. The overall best performing model, based on an ANN, has a good prediction performance together with low computational and architectural complexities (evaluated on the basis of the NetScore metric), making its deployment on an edge device feasible.

## 1. Introduction

The introduction of Information and Communication Technologies (ICTs) in the agricultural sector, aiming to improve productivity and sustainability, is currently a well-established practice. Indeed, the integration of heterogeneous technologies such as, just to name a few, Internet of Things (IoT) and Machine Learning (ML), allows us to simplify and enhance the management of the so-called Smart Farms [1]. Due to this trend of technological transformation, usually denoted as Smart Agriculture (SA) or Smart Farming (SF), in recent years, greenhouses’ productive processes have been optimized, for example in terms of increasing automatization.

To this end, greenhouses play a crucial role in worldwide agricultural productions. Indeed, through the creation of optimal growing conditions for indoor cultivation [2], vegetables, fruits, herbs and other kinds of edible products can be farmed anytime and everywhere, regardless of their seasonality or the (eventually adverse) weather conditions of their growing area. Therefore, the adoption of greenhouses offers various advantages, such as the extension of the cultivation periods of seasonal crops, the local production of non-endemic food, and reduced resource consumption, in terms of water, land and pesticides [3].

One of the most relevant (and challenging) aspects of greenhouse management is the development and maintenance of suitable growing habitats for the inside plants. This internal habitat, also referred to as “inner climate” or “micro-climate,” corresponds to the complex set of environmental variables internal to the greenhouse (e.g., soil moisture, air humidity and temperature, and solar radiation) affecting the inner products’ growth and depends on several interrelated elements [4]. In detail, there are (i) *internal* factors, such as, for example, the greenhouse’ size and the cooling, warming or ventilation systems installed internally (thus referring to the greenhouse’s actuators), and (ii) *external* factors (or “variables”), such as weather meteorological conditions (e.g., wind speed, solar radiation, temperature, and humidity) [5].

Nowadays, monitoring and controlling a greenhouse’s micro-climate has been simplified and automatized thanks to the integration of heterogeneous technologies, which can be grouped into three categories.

- *First*, (sensor) data related to relevant environmental variables internal to the greenhouse, which have to be maintained within suitable ranges (e.g., air humidity and temperature), are collected through devices equipped with sensors (denoted as IoT sensing nodes, or sensor nodes, SNs), generally organized as Wireless Sensor Networks (WSNs). Moreover, internal greenhouse data gathered by SNs are usually sent to less constrained nodes, denoted as gateways (GWs) and connected to the Internet. GWs forward SNs’ data to processing and storing infrastructures located in the Cloud [6]. Then, data can be retrieved and visualized (through appropriate User Interfaces, UIs), as well as kept as input data for further processing. Hence, monitoring of relevant variables inside the greenhouse is relevant for both end-users (farmers) and for researchers [7,8,9,10,11].
- *Secondly*, additional control devices (i.e., actuator nodes), installed inside the greenhouse in order to regulate its internal climate [12,13], can be integrated within the aforementioned collection system. As an example, if a dangerous air humidity index is detected by SNs, a ventilation system would automatically be activated in order to lower the air humidity.
- *Thirdly*, complex models and/or forecasting algorithms are developed with the goal of predicting the future values of the monitored environmental variables, for example allowing us to preemptively schedule some operations (e.g., the activation of a warming system) to avoid these internal variables reaching undesired conditions (i.e., too low temperatures). To this end, the greenhouse’s internal variables have been satisfactorily forecast through Deep Learning (DL) algorithms, e.g., based on Neural Networks (NNs) [14,15,16], and selecting data collected from different sources as input (namely, internal and external variables of a greenhouse, possibly measured by SNs).

For the sake of completeness, NN-based prediction algorithms can also be employed to infer missing sensor data, such as those not properly gathered inside the greenhouse due to a temporary lack of network connectivity, as well as to maintenance operations.

Since the adoption of NN-based algorithms generally provides significant benefits, they are more and more often integrated with IoT technologies in the context of SA. Moreover, although in recent years, these algorithms are mainly intended to be executed in the Cloud, a new promising IoT trend, denoted as EdgeAI and related to the transfer of intelligence (e.g., AI algorithms) from the Cloud down to the Edge (e.g., on IoT edge devices), is emerging [17]. Indeed, the execution of AI algorithms on IoT edge devices, which are located near the data sources (namely, SNs), provides relevant advantages, such as the reduction of the amount of data to be forwarded by IoT edge devices to the Cloud, thus reducing the network load, lowering the response latency, and, finally, supporting scalability. As a drawback, algorithms executed on-the-Edge have to be designed taking into account the limitations imposed by constrained IoT devices, with memory footprint, computational, and energy resources significantly lower than those offered by Cloud platforms. Thus, while modeling EdgeAI algorithms, there is the need to look for a balanced trade-off between reaching the best prediction performance and implementing a prediction model computationally “lightweight enough” to be executed by a constrained device (e.g., the required memory occupation has to be compatible with the one available on the device). In order to quantify the trade-off involved in achieving the two contrasting goals, in [18] the authors propose the NetScore metric, which is intended as a quantitative relative assessment of the prediction performance with computational and architectural complexities of a Deep NN (DNN).

In order to improve the management of greenhouses and to control their internal variables, in [19]—being the paper “AI at the Edge: a Smart Gateway for Greenhouse Air Temperature Forecasting” presented in the IEEE International Workshop on Metrology for Agriculture and Forestry (MetroAgriFor)—the authors have presented a novel approach based on the joint adoption of NNs, Edge Computing, and IoT technologies. In detail, the authors have “enriched” an IoT device, denoted as Smart GW and acting as a GW for a WSN installed in a greenhouse, in order to: (i) monitor its inner air temperature through an “edge intelligent” approach; (ii) locally forecast future inner air temperature’s values; and (iii) regulate, according to the obtained results, the greenhouse air temperature (through internal actuators). From an algorithmic point of view, the authors have designed a lightweight device-friendly forecasting model, based on a fully-connected Artificial NN (ANN), able to predict the air temperature inside a greenhouse, also on the basis of the outside weather conditions.

In order to extend the solution proposed in [19], in the current paper, the authors evaluate an alternative approach to build an air temperature forecasting model to be executed on the Smart GW presented in [19] (with similar purposes). In particular, rather than relying on meteorological weather conditions (collected outside the greenhouse), the aim is now on the exploitation of air temperature values collected inside the greenhouse to forecast the air temperature evolution. Moreover, the authors evaluate the performance (in terms of prediction precision and lightweightness) of the model developed following a novel approach, denoted as *time series-oriented*, with respect to the model proposed in [19]. Then, the authors investigate how the model’s parameters—namely, the number of input variables (i.e., air temperature values), their sampling interval, and the NN architecture—influence the model’s prediction performance. In detail, the following three types of NN architectures (which, according to the literature, outperform all other types of NNs for time series-based forecasting) have been evaluated: ANN, Recurrent NNs (RNNs), and Long Short-Term Memory (LSTM) networks. Finally, a comparison between the developed models and relevant approaches proposed in the literature for the same task (namely, air temperature forecasting inside greenhouses) is performed, in terms of prediction performance considering three metrics widely adopted in regression problems: (i) Root Mean Squared Error (RMSE); (ii) Mean Absolute Percentage Error (MAPE); and (iii) coefficient of determination (R${}^{2}$). Unlike the typical approach followed in the literature, the authors also focus on the performance analysis in relative terms with respect to computational and architectural complexities. More precisely, this goal is achieved by relying on the use of the NetScore metric (in order to evaluate the complexity of the NN-based models to be run on constrained devices)—to the best of the authors’ knowledge, this has never been considered before. This is fundamental in order to identify the proper model for IoT edge devices, but seldom discussed in the literature in the context of greenhouses’ internal variables forecasting.

**Figure 1:**
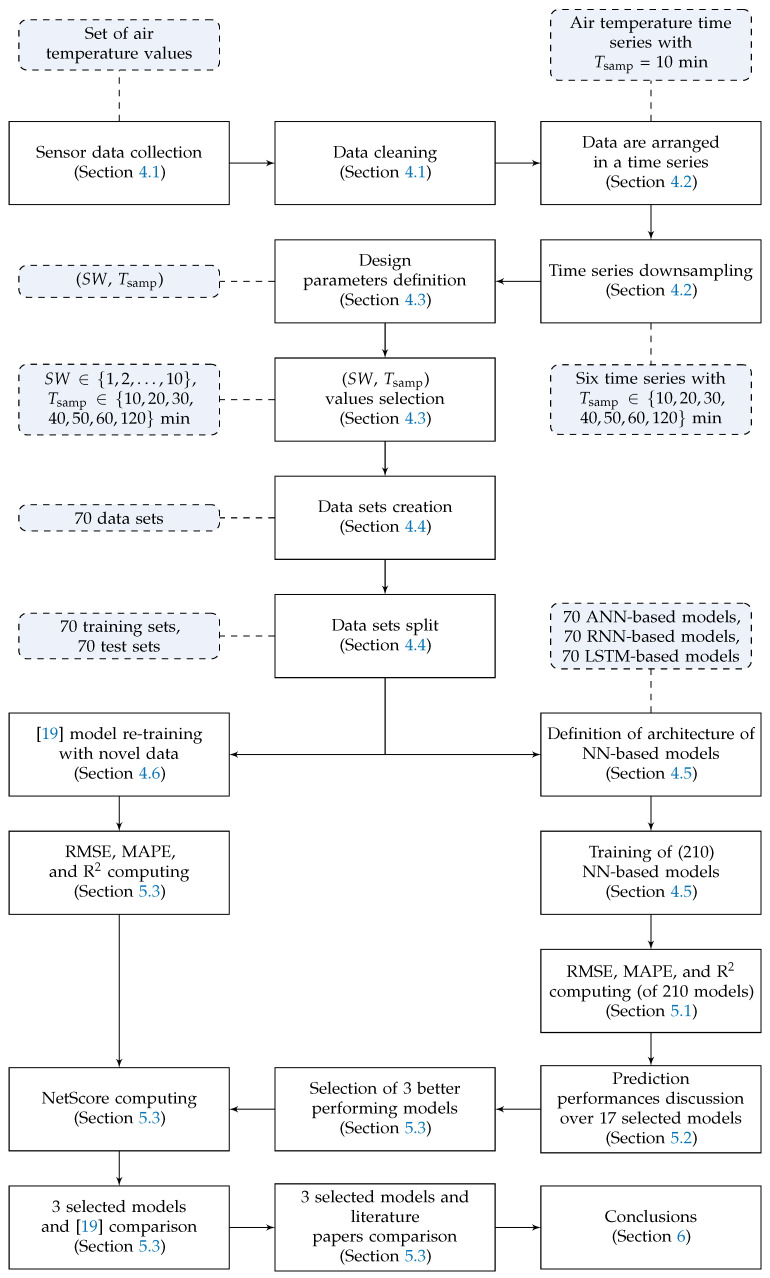
Methodological steps (white rectangles) and corresponding outcomes (violet rectangles with rounded corners) of this paper, with reference to its internal structure.

The rest of the paper is organized as follows. Section 2 gives an overview of NNs and an evaluation metrics adopted in this paper. In Section 3, a review on literature works is presented, while in Section 4 the adopted methodology is described. In Section 5, the experimental results obtained with the proposed models are outlined and discussed. Finally, in Section 6 some conclusions are drawn. To conclude, the methodological steps followed in this manuscript to perform target analysis and select the proper EdgeAI algorithm (to be deployed on the Smart GW) are summarized in Figure 1, with reference to the paper’s internal structure.

## 2. Background

### 2.1. Overview on Neural Networks

In general terms, NNs can be seen as the “backbone” required to build ML-oriented algorithms that start from a set of data (or data set) and fit them into a parametric model [20,21,22], in order to solve different tasks, such as prediction [23]. More precisely, the parameters of these models are learnt from a group of samples—composing a training set—in a (preliminary) training phase, while their effectiveness is assessed on a samples test set in a (consecutive) test phase, adopting one or more evaluation metrics (as will be discussed in Section 2.2).

Due to their ability to (i) discover complex relations among data, (ii) be robust against data uncertainty, and (iii) predict output variables’ values almost in real-time, NNs are appealing in many application areas, including greenhouse’s inner variables’ forecasting. As a drawback, in order to obtain an accurate forecasting model, NNs normally require a sufficiently large number of available data (sometimes not easy to be gathered) representative of the model to build.

Despite several types of NN-based architecture proposed in the literature (differing in terms of building elements, interconnections, and learning algorithms), a simple type is given by the Multi-Layer Perceptron (MLP) NN [24], in the following simply denoted as ANN. In detail, the model is built on top of multiple and interconnected processing units, denoted as *neurons* (or simply network nodes), organized in *layers*. Then, its internal organization allows input data to be processed from the first stages of the network to the final ones, thus allowing information to flow across the network, producing output variables. Moreover, models in which the data stream inside the network is linearly processed from the first layer (denoted as input layer) through one or more internal layers (denoted as hidden layers), till the final layer (denoted as output layer), are labeled as feed-forward. Furthermore, in the case that each node of a network layer is connected with every node of the previous layer, the network is denoted as fully-connected. Although an exhaustive discussion of the training process of a NN (from a mathematical point of view) is out of the scope of this paper, in the following the authors provide some high-level considerations, especially regarding the structure of a fully-connected feed-forward ANN.

**Figure 2:**
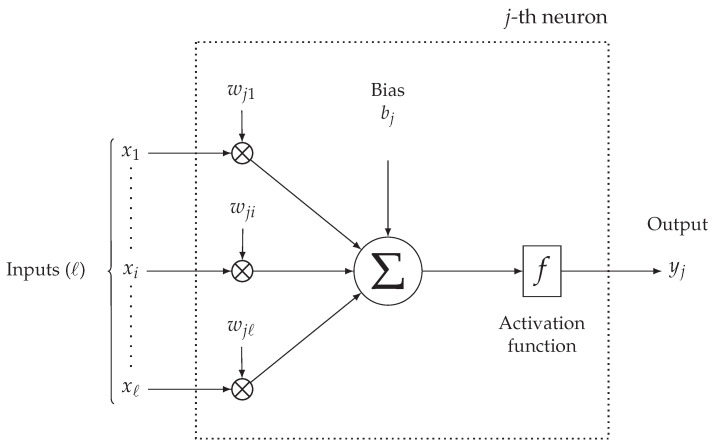
Simplified mathematical model of the processing of the *j*-th neuron inside a MLP ANN.

Each neuron of the network (except for the input layer) receives information from all neurons in the previous layer (or from a subset, if the ANN is not fully-connected). In Figure 2, a simplified mathematical model describing the *j*-th neuron of an intermediate layer is shown. The messages arriving from the *ℓ* connected neurons from the previous layer, denoted as ${\left\{x_{i}\right\}}_{i=1}^{\ell }$, are combined within the *j*-th neuron through proper parametric functions, the most popular one being based on a weighted sum, with weights ${\left\{w_{ji}\right\}}_{i=1}^{\ell }$, of the input messages. In detail, each *j*-th neuron’s input $x_{i}$ is multiplied by a weight $w_{ji}$ whose value is learnt during the ANN training phase (thus not *a-priori* fixed) and, then, added to the other weighed inputs messages. Then, a bias term $b_{j}$ is added to the sum. Finally, before being forwarded to the next ANN’s layer, the output of a neuron is processed by a non-linear activation function, denoted as f(·): a relevant example is the Rectified Linear Unit (ReLU) function f(x)=max(0,x) [21]. The overall output of the *j*-th neuron, denoted as $y_{j}$, can then be expressed as follows:

(1) $$\begin{array}{ccc} y_{j} & = & f\left(\sum_{i=1}^{\ell }w_{ji}\;x_{i}+b_{j}\right). \end{array}$$

The training process of a NN is usually pursued through several rounds in which, according to a cost function quantifying the similarity between the NN’s outputs and its desired values, the weights (in other words, the model’s parameters) are updated to improve the performance or, more precisely, to reduce a cost function. To this end, there exist several algorithms implementing the learning process, among which one of the most popular ones is Back Propagation (BP) with gradient descent [23].

**Figure 3:**
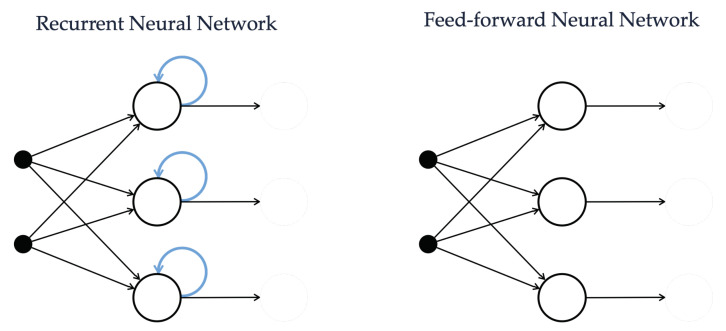
Comparison between the internal organization of a RNN, owing a sort of internal memory, and an ANN.

In the context of variable prediction in a greenhouse, beside ANNs, RNN and LSTM networks have been successfully applied to forecast air temperature (as detailed in Section 3). Indeed, due to their ability in discovering a connection between temporally-close data, RNNs and LSTM networks are in general valid alternatives for solving time series-based forecasting problems. From a high-level perspective, this is mainly due to the fact that, for each stage in the training phase, a simple RNN can remember its internal state (namely, the output values of each of its neurons, as outlined in Figure 3) and exploit this state to compute the next state in the following round of training. Therefore, this internal organization allows us to analyze data in a recurring fashion, performing loops on data and resulting in a sort of short-term memory.

In order to overcome some limitations of RNNs, such as a vanishing gradient with long time series, which makes it difficult to learn long-term dependencies from data using RNN [25], valid alternatives are LSTM networks [26] (or simply LSTMs). From a conceptual point of view, LSTMs are built around a gated cell in which weights, learned during the training phase, define which information is more relevant to remember, thus allowing us to decide which value needs to be stored inside the (memory of the) cell and which one needs to be discarded. This allows us to select which information provided by the previous network’s training round should be kept and which should not, better highlighting long-term relations among data.

### 2.2. Evaluation Metrics

The quality of a forecasting model, evaluated in terms of prediction performance in a comparative way with respect to multiple models devoted to the same task, is usually quantified selecting one or more proper metrics. In regression problems, such as the forecasting of future air temperature values inside a greenhouse, widely considered evaluation metrics are RMSE, MAPE, and R${}^{2}$ [21], defined as follows:

(2) $$\begin{array}{ccc} \mathrm{RMSE} & = & \sqrt{\frac{\sum_{j=1}^{n}{\left(d_{j}-p_{j}\right)}^{2}}{n}} \end{array}$$

(3) $$\begin{array}{ccc} \mathrm{MAPE} & = & \frac{1}{n}\sum_{j=1}^{n}\left|\frac{d_{j}-p_{j}}{d_{j}}\right|\times 100 \end{array}$$

(4) $$\begin{array}{ccc} R^{2} & = & {\left[\frac{\sum_{j=1}^{n}\left(d_{j}-\bar{d}\right)\left(p_{j}-\bar{p}\right)}{\sqrt{\sum_{j=1}^{n}\left(d_{j}-\bar{d}\right)\sum_{j=1}^{n}\left(p_{j}-\bar{p}\right)}}\right]}^{2} \end{array}$$

where *n* is the number of predicted (and corresponding actual) samples; $\left\{d_{j}\right\}$ and $\left\{p_{j}\right\}$ are the actual and forecast values of the *j*-th sample of the phenomenon under observation (e.g., the temperature), respectively; and $\bar{d}≜\left(\sum_{j=1}^{n}d_{j}\right)/n$ and $\bar{p}≜\left(\sum_{j=1}^{n}p_{j}\right)/n$ are the average values of the actual and forecast samples, computed as arithmetic averages over all the *n* actual and forecast samples, respectively.

The RMSE, defined according to Equation (2), represents the standard deviation of the difference between the actual values (to be predicted) and the values predicted by the model (or residuals). The closer to zero the RMSE is, the better the algorithm performs.

The MAPE, defined as in Equation (3), is a percentage quantifying a relative distance between predicted and actual values. As for the RMSE, the closer to zero the MAPE distance, the better the performance.

Finally, R${}^{2}$, defined according to Equation (4), is a normalized (between 0 and 1) statistical metric quantifying the adaptability of a regression model. The closer to one R${}^{2}$ is, the more accurate the prediction model is.

A recently proposed metric, relevant for the evaluation of a NN N to be run on an edge device, is the NetScore metric [18], denoted as Ω and expressed as follows:

(5) $$\begin{array}{ccc} \Omega (N) & = & 20\log\left(\frac{a{\left(N\right)}^{\alpha }}{p{\left(N\right)}^{\beta }\times m{\left(N\right)}^{\gamma }}\right) \end{array}$$

where a(N) is the accuracy of the model (i.e., the correct prediction rate in a classification task); p(N) is the number of parameters of a NN, also denoted as architectural complexity; m(N) is the number of Multiply–ACcumulate (MAC) operations performed during NN inference, also denoted as computational complexity; and α,β,γ are coefficients which allow to control the relative weights (for the purpose of NetScore evaluation) of accuracy, architectural complexity, and computational complexity of the model N, respectively.

As can be seen from Equation (5), the NetScore metric assigns to an NN a score that is logarithmically proportional to a ratio between prediction accuracy and complexity. This means, for example, that models with high accuracy and a small number of parameters have a high NetScore value; on the contrary, models with moderate accuracy but high complexity have a small NetScore value. Furthermore, models with different accuracy, number of parameters, and MAC operations may have comparable values in terms of the NetScore metric.

Finally, the relative impact of the components of the logarithmic ratio of the NetScore (namely, accuracy, architectural and computational complexities) are regulated by the exponential coefficients α, β, and γ, which, in turn, allow us to adjust the weight of each factor according to its practical relevance for the application at hand. As an example, higher (than default) values can be assigned to β and γ whenever the NN model needs to be extremely simple in terms of computational and architectural complexities. On the other hand, if the model’s accuracy is the most relevant algorithmic feature, then α can be set to a high value. In general, for the majority of the applications, it is possible to set α, β, and γ equal to 2, 0.5 and 0.5, respectively [18]). This means that, from a general point of view, the relevance of the model’s accuracy is usually higher than the (architectural and computational) complexity of the model itself.

## 3. Related Work

In Table 1, relevant literature references are summarized, which are commented in more detail below. As outlined in Section 1, the joint adoption of IoT and ML in the area of SA allows to simplify the maintenance of a greenhouse micro-climate, thus automatizing the control of its inner variables. Usually, this challenging and complex goal is pursued through three main steps. *First*, a WSN is deployed inside the greenhouse in order to collect and monitor a subset of relevant environmental parameters (namely, the greenhouse internal variables) [7,9,11]. *Second*, one or more control systems, based on devices that can control the greenhouse’s actuators, are introduced in order to automatically maintain a proper internal climate [12,13]. *Third*, the internal variables’ future trends are forecast with prediction techniques, based on ML algorithms, such as NNs [3,14,15,16,27,28].

In the context of greenhouse air temperature forecasting, NNs, due to their ability in learning patterns from data related to non-linear systems without an a priori knowledge of the system model [28], have become an extremely popular alternative to more consolidated techniques—e.g., physical methods based on mathematical theory and black-box approaches based on modern computational technology (e.g., Particle Swarm Optimization, PSO) [4].

As shown in Table 1, several algorithms to forecast the air temperature with NN-based models have been proposed. Although aiming at the same purpose, models in Table 1 differ in terms of adopted input variables, NN architecture, and data sources. Indeed, input parameters can include variables related to the surrounding environment external to the greenhouse, as well as environmental variables internal to the greenhouse, or a combination of them. Usually, the first group of variables cannot be controlled but influence the internal climate of the greenhouse: this is the case, for example, of external temperature, solar radiation, humidity, and wind speed [3,14,15,27]. In most cases, the second group of variables can instead be influenced by activating (or deactivating) some actuators installed inside the greenhouse: this is the case, for example, of internal air temperature and humidity, soil moisture, and CO${}_{2}$ [14,16,28]. As outlined in Table 1, the main types of NN architectures discussed in the literature, and achieving satisfactory prediction performance, are ANN, RNN, LSTM and Radial Basis Function (RBF) networks.

Considering the principles on the basis of their designs, it is possible to conceptually separate literature models into the following categories: (i) time series-oriented approaches, (ii) “pure” ML approaches, and (iii) hybrid approaches. In detail, the first type of approaches solves the task of predicting future air temperature values as a time series forecasting problem, leveraging the following features characterizing time series data, i.e., data which are periodically sampled and have a time reference (as sensor data): trends, seasonality, and correlation between samples which are close in time. The time series peculiarity of having, in most cases, temporally-close data linked by a relation, can be successfully discovered by NN models, such as RNNs [27] and LSTM networks [28], and exploited in order to achieve prediction performance better than other types of NN architectures. The use of data coming from other data sources, without taking into account the relation existing between temporally-close data of the same time series, can also be valuable in order to forecast air temperature. These sources can include data related to external or internal variables to the greenhouse, correlated to the air temperature values to be predicted. With this approach, the use of RBF networks [15,16] and ANNs [29] have been very successful in air temperature forecasting. To conclude, the two approaches can be integrated leading to a hybrid approach, selecting the NN architecture which best suits the prediction problem at hand [3].

The authors now comment briefly on the limitations of existing literature and on the steps forward proposed in the current paper. In [3], an air temperature forecasting model based on an ANN, predicting air temperature with a RMSE of 2.5÷3.0 ${}^{\circ }C$, has been presented. Better results, in terms of RMSE, have been achieved with the same type of NN architecture (namely, ANN) in [14,19], reaching a RMSE equal to 0.839 ${}^{\circ }C$ and 1.50 ${}^{\circ }C$, respectively. These results can be eventually justified by the fact that the last two models have been trained with a wider data set (in other words, a data set with a number of samples 4 or 64 times greater with respect to the one available in [3]). In [27], a RNN-based model has been built using a data set of comparable size to [3] but achieving a better RMSE (equal to 0.865 ${}^{\circ }C$) than [19,27], but lower than [14]. Moreover, RBF networks have been adopted in [16] to build a model which outperforms all the above-mentioned papers in terms of RMSE. Unlike these literature works, in which the forecasting model designs take into account only one type of NN architecture, in [15,28], more than one type of NN architecture have been compared in order to perform a more comprehensive analysis. The prediction performance of the models presented in [15,28] is comparable with that in [14,27], better than that in [3,19], but lower than that in [16].

The above-cited works (also outlined in Table 1) do not provide any detail concerning the computational and architectural complexities of their proposed models. Instead, such complexities are key factors while designing IoT applications in which NN-based algorithms are deployed on embedded devices (usually located on the network’s edge). Indeed, since most literature works—showing remarkable results in the air temperatures forecasting (e.g., with a RMSE value lower than 1 ${}^{\circ }C$)—are intended to be executed in the Cloud and not on IoT (edge) devices, no discussion concerning the proposed model’s complexity is provided, as this aspect is not a limitation. However, since the diffusion of EdgeAI (due to its significant advantages) is rapidly growing, taking into account the complexity, while deploying new algorithms for IoT applications (running on edge devices), is extremely relevant. In the above-cited works, this aspect has not been exhaustively discussed in the context of forecasting air temperature within greenhouses. Filling this literature gap is one of the aims of the current paper.

To conclude, in a previous work [19], the authors have discussed the design of an edge device-friendly air temperature forecasting model following the pure ML approach (the second approach previously introduced). More precisely, in order to forecast future values of air temperature inside a greenhouse in a demonstrator of the H2020 project AFarCloud [30], the authors have selected the external weather conditions as input variables for the model. Moreover, the authors have implemented an algorithm which, due to its low computational and architectural complexities, can be easily run by an edge device (i.e., the Smart GW) and can predict future air temperatures with performance results comparable to the literature. As anticipated at the end of Section 1, another goal of this paper is to experimentally investigate a novel time series-based approach in a comparative way with respect to [19] and existing literature approaches, including a comprehensive analysis of the prediction performance in relative terms with respect to the (computational and architectural) complexity of the developed model. In particular, the authors will show that their model, even with limited complexity, incurs minimal (or no) performance degradation with respect to Cloud-oriented approaches.

## 4. Methodology

As outlined in Section 1, the aim of this paper is three-fold. *First*, the authors want to evaluate an alternative approach to solve the ML-oriented task presented in [19], namely the deployment of an “edge device-friendly” air temperature forecasting model (intended to be run on a Smart GW). Instead of the weather conditions external to the greenhouse, the NN-based model deployed with the novel approach (i.e., time series-oriented, as discussed in Section 3) takes as input variables only air temperature values collected inside the greenhouse. Three architectural types of NN are considered: ANN, RNN, and LSTM. The authors analyze the impact, on the prediction performance of the selected three architectures, of two design parameters: (i) the number of model’s input variables (or, in other terms, the size of the data sliding window used by the model), denoted as SW, and (ii) their sampling period, denoted as $T_{\mathrm{samp}}$ (dimension: [min]). *Second*, the model presented in [19] has been re-trained with data collected from August 2019 to the end of November 2020, to make the data set coherent with that available for the new approach (in [19], the data set was composed by data collected from August 2019 to the end of May 2020). *Third*, the experimental performances of the proposed models have been compared with those outlined in Table 1.

From a methodological point of view, the following steps are undertaken.

1. Relevant air temperature data, measured with sensors inside a greenhouse associated with an Italian demonstrator of the H2020 project AFarCloud [30], are collected and processed to remove outliers and spurious data (Section 4.1).
2. The greenhouse indoor temperature sensor data collected with a sampling period $T_{\mathrm{samp}}=10$ min are arranged in a time series. Furthermore, from this original time series, six additional time series are derived downsampling the first time series with longer sampling periods (Section 4.2).
3. The number of input variables of the model SW and the sampling period $T_{\mathrm{samp}}$ are defined as the two design parameters. Moreover, $T_{\mathrm{samp}}$ is reintegrated as the prediction time horizon; in fact, the predicted temperature value is the one corresponding to the next temperature value after the most recent one of the sliding window: this samples is, by construction, $T_{\mathrm{samp}}$ ahead. Furthermore, a proper set of values related to these parameters is selected for testing purposes (Section 4.3).
4. Starting from the collected sensor data and according to the number of parameters’ values to be tested, multiple data sets are created. Furthermore, each data set is split into training and test subsets (Section 4.4).
5. Three NN architectures, based on an ANN, a RNN, and a LSTM, are introduced and trained with the data sets resulting from the previous steps (Section 4.5).
6. The NN model presented in [19] is re-trained with a significantly larger data set—including data from 6 more months (Section 4.6).
7. All models are evaluated on the test subsets and their performances are compared in terms of RMSE, MAPE, R${}^{2}$, and NetScore (Section 5).
8. Finally, the best three models (among a total of 210) on the considered engineered data sets (step 4) are performance-wise compared with relevant literature approaches (Section 5).

### 4.1. Data Collection and Cleaning

**Figure 4:**
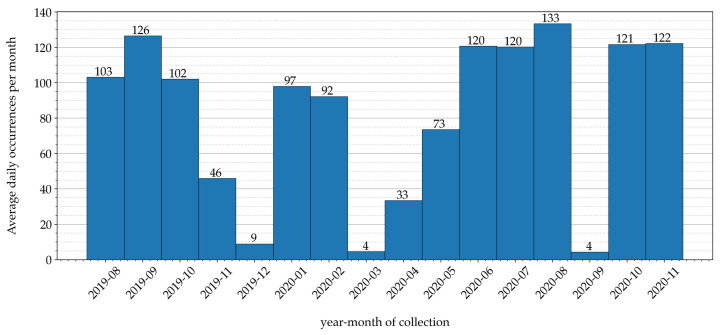
Sensor data collected during a 16-month time period: in each month, the average daily number of collected samples (obtained as the ratio between the number of gathered samples per month normalized and the number of days of the month) is shown.

Sensor data related to air temperatures have been gathered inside the greenhouse of an Italian farm, denoted as Podere Campàz [31], through the LoRaFarM platform, a Farm-as-a-Service (FaaS) architecture proposed in [8]. In detail, air temperature values have been measured with a sampling interval of 10 min, for a time period of 16 months—from the beginning of August 2019 to the end of November 2020. The distribution of collected data, over the 16-month time period, is shown in Figure 4: in each month, the authors indicate the average (over the month) number of data collected daily.

Analyzing the data distribution shown in Figure 4, it can be seen that data have been irregularly collected across the months of the collection period. This is due to various reasons: for example, during December 2019, March 2020, and September 2020, the data irregularity was caused by a temporary Internet connectivity loss, which prevented collected data from being forwarded, through the LoRaFarM platform, to a storage repository placed in the Cloud.

On the other hand, the samples’ distribution over actual collection daily hours has an almost perfect uniform trend, meaning that the number of data collected during different hours of the day in the 16-month time period is approximately the same at each hour. Indeed, the overall amount of “raw” data gathered during this stage correspond to $N_{\mathrm{samp}}$ = 40,033 samples, with the number of samples collected per hours varying from a minimum of 1649 samples to a maximum of 1694 samples, with an average value of 1668 samples and a standard deviation of approximately 11 samples.

### 4.2. Engineering Time Series from Sensor Data

**Figure 5:**
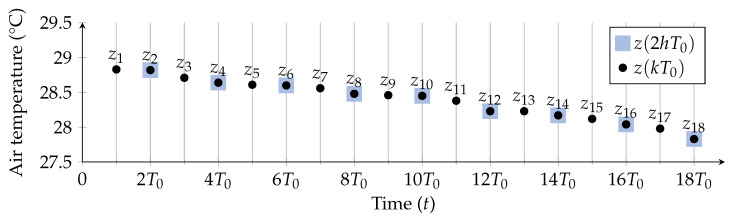
Illustrative representation of the first 18 samples ${\left\{z_{k}^{\left(T_{0}\right)}=z\left(kT_{0}\right)\right\}}_{k=1}^{18}$ (black dots) and of the first 9 samples ${\left\{z_{h}^{\left(2T_{0}\right)}=z\left(2hT_{0}\right)\right\}}_{h=1}^{9}$ (black dots over violet squares), obtained downsampling $\left\{z_{k}^{\left(T_{0}\right)}\right\}$ with a factor equal to 2.

As mentioned in Section 4, sensor data were collected with a (real) sampling period $T_{\mathrm{samp}}=T_{0}=10$ min. The corresponding time series, describing the measured air temperature inside the greenhouse during the considered period of 16 months, is denoted as ${\left\{z_{k}^{\left(T_{0}\right)}\right\}}_{k=1}^{N_{\mathrm{tot}}}$, where *k* corresponds to the time instant $kT_{0}$—denoting $T_{0}$ (with k=1) as the instant of collection of the first sample and $N_{\mathrm{tot}}T_{0}$ (with $k=N_{\mathrm{tot}}$) as the instant of collection of the last sample—or, with a more compact notation, simply $\left\{z_{k}^{\left(T_{0}\right)}\right\}$. More precisely, denoting the temperature in the greenhouse as z(t), the time series’ sample $z_{k}^{\left(T_{0}\right)}=z\left(kT_{0}\right)$ corresponds to the air temperature measured at time instant $t=kT_{0}$. As an additional clarification, in this paper a deterministic temperature signal z(t) has been considered, as it refers to the specific real data collected from sensors. In order to generalize this approach, the air temperature should be modeled as a stochastic process Z(t). However, this goes beyond the scope of this paper. In Figure 5, an illustrative representation of the first 18 samples of the time series $\left\{z_{k}^{\left(T_{0}\right)}\right\}$ (black dots), denoted as $\left\{z_{k}\right\}$ for simplicity, is shown.

The authors remark that, because of the methodology selected to gather sensor data inside the greenhouse, some samples of the time series $\left\{z_{k}^{\left(T_{0}\right)}\right\}$ may not be available—i.e., the LoRaFarM platform may suffer from temporary lack of Internet connectivity and, thus, lose some sensor data. Hence, this means that there exist $N_{\mathrm{lost}}$ instants of time (${\hat{k}}_{1}T_{0},\ldots ,{\hat{k}}_{N_{\mathrm{lost}}}T_{0}$) at which the values of air temperature ${\left\{z_{{\hat{k}}_{i}}^{\left(T_{0}\right)}\right\}}_{i=1}^{N_{\mathrm{lost}}}$ are not available—for example, one can set $z_{{\hat{k}}_{i}}^{\left(T_{0}\right)}=$ NaN. Moreover, for this reason, the overall number of samples in the time series, denoted as $N_{\mathrm{samp}}$, is smaller than the total number of samples $N_{\mathrm{tot}}$ ideally collected in the considered 16 month time interval with a sampling period equal to $T_{0}$ = 10 min and no data loss—more precisely, $N_{\mathrm{tot}}$ = $N_{\mathrm{samp}}+N_{\mathrm{lost}}$.

Focusing on downsampling, it is useful to reduce the number of samples in a time series in the context of time series’ forecasting and analysis, indicating with λ the downsampling factor, the downsampled time series can be expressed as follows:

(6) $$\;{\left\{z_{h}^{\left(\lambda T_{0}\right)}=z\left(\lambda hT_{0}\right)\right\}}_{h}={\left\{z_{\lambda h}^{\left(T_{0}\right)}\right\}}_{\lambda h}\;h=1,2,\ldots$$

To this end, in Figure 5 the downsampled version (with λ=2) of the original time series is shown, with the corresponding samples identified by circles with squares.

In order to evaluate how the sampling period $T_{\mathrm{samp}}$ influences the prediction performance of the model, 6 additional time series are built downsampling the original time series $\left\{z_{k}^{\left(T_{0}\right)}\right\}$ with $T_{\mathrm{samp}}$ equal to $2T_{0}=20$ min, $3T_{0}=30$ min, $4T_{0}=40$ min, $5T_{0}=50$ min, $6T_{0}=60$ min, and $12T_{0}=120$ min, i.e., by setting the downsampling factor λ to 2, 3, 4, 5, 6, and 12, respectively. The corresponding time series are denoted as $\left\{z_{k}^{\left(2T_{0}\right)}\right\}$, $\left\{z_{k}^{\left(3T_{0}\right)}\right\}$, $\left\{z_{k}^{\left(4T_{0}\right)}\right\}$, $\left\{z_{k}^{\left(5T_{0}\right)}\right\}$, $\left\{z_{k}^{\left(6T_{0}\right)}\right\}$, $\left\{z_{k}^{\left(12T_{0}\right)}\right\}$, respectively.

All the created time series have been used to build a total of 70 different data sets (i.e., 10 data sets for every time series, with a different number of input variables each), as detailed in Section 4.4. Considering these time series allows to determine the best sampling period for the phenomenon of interest (inner air temperature).

### 4.3. Sliding Window-Based Prediction

**Figure 6:**
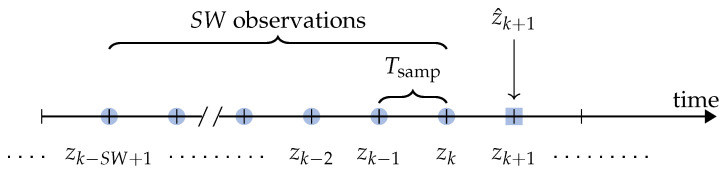
Sliding window-based prediction at epoch *k*: the SW observations ${\left\{z_{k-i+1}\right\}}_{i=1}^{SW}$ are used to predict the value $z_{k+1}$, denoted as ${\hat{z}}_{k+1}$—the superscript ${}^{\left(T_{\mathrm{samp}}\right)}$ is omitted for simplicity.

As described in Section 4, the prediction approach is based on the use of a sliding window. More precisely, the authors consider SW consecutive samples of the target time series, with sampling interval $T_{\mathrm{samp}}$, to predict the value of the next sample. With reference to Figure 6, at epoch *k*:

(7) $${\hat{z}}_{k+1}^{\left(T_{\mathrm{samp}}\right)}={\hat{z}}_{k+1}^{\left(T_{\mathrm{samp}}\right)}\left(z_{k-SW+1}^{\left(T_{\mathrm{samp}}\right)},\ldots ,z_{k}^{\left(T_{\mathrm{samp}}\right)}\right),$$

where ${\hat{z}}_{k+1}^{\left(T_{\mathrm{samp}}\right)}$ corresponds to the predicted value of the (true) sample $z_{k+1}^{\left(T_{\mathrm{samp}}\right)}$ and, at the right side, the dependency from the previous samples is highlighted.

Since the parameters SW and $T_{\mathrm{samp}}$ influence the prediction performance of the model, they need to be optimized. For this reason, different combinations of SW and $T_{\mathrm{samp}}$ are considered, comparing the corresponding prediction performance. In detail, 10 values of SW (namely, from 1 to 10 samples) and, as described in Section 4.2, 7 values for $T_{\mathrm{samp}}$ (namely, 10, 20, 30, 40, 50, 60 and 120 min) are considered.

For the sake of clarity, it is noteworthy to remark that, since SW corresponds to the input size of a model (the parameter *ℓ* in Figure 2 for a neuron of the input layer), at the input of the model there is a SW-dimensional vector of SW features corresponding to air temperature values sampled with a fixed period $T_{\mathrm{samp}}$ and, thus, coming from the time series $\left\{z_{k}^{\left(T_{\mathrm{samp}}\right)}\right\}$ built in the previous stage. As an example, a model built selecting SW=4 and $T_{\mathrm{samp}}=10$ min is fed with input vectors having 4 features corresponding to 4 consecutive air temperature samples collected every $T_{\mathrm{samp}}=10$ min.

### 4.4. Data Pre-Processing and Data Sets Creation

Since the authors want to evaluate the prediction performance of the model adopting different combinations of $\left\{SW,T_{\mathrm{samp}}\right\}$ pairs, multiple data sets to train and test the algorithm can be engineered. More precisely, 70 data sets have been created, each one associated with a specific $\left\{SW,T_{\mathrm{samp}}\right\}$ pair, where SW∈{1,2,…,10} and $T_{\mathrm{samp}}\in \left\{10,20,30,40,50,60,120\right\}$ min. In detail, using a compact notation, the authors denote as $D_{SW}^{\left(T_{\mathrm{samp}}\right)}$ the data set obtained with the corresponding values of $T_{\mathrm{samp}}$ and SW. Each entry of the data set is a vector $\underline{d}\left(k\right)$ of true temperature values defined as follows:

(8) $$\underline{d}\left(k\right)≜\left(z_{k-SW+1}^{\left(T_{\mathrm{samp}}\right)},\cdots ,z_{k}^{\left(T_{\mathrm{samp}}\right)},z_{k+1}^{\left(T_{\mathrm{samp}}\right)}\right).$$

**Figure 7:**
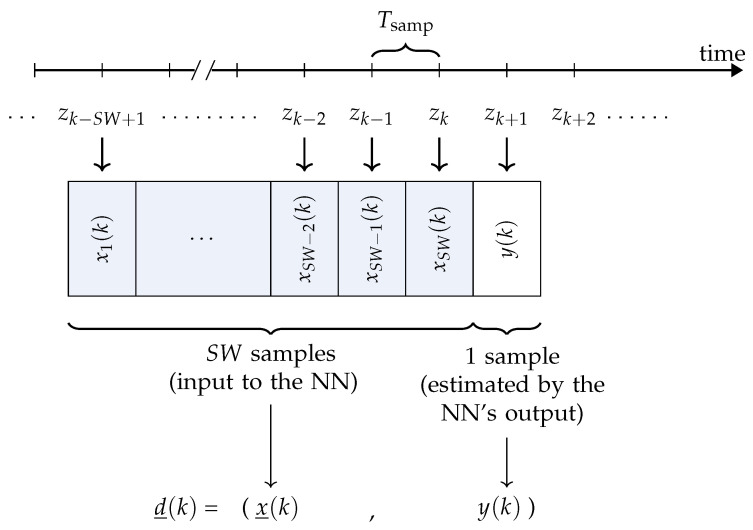
The *k*-th sample $\underline{d}\left(k\right)$ in the data set $D_{SW}^{\left(T_{\mathrm{samp}}\right)}$ is composed of SW values of air temperatures (composing the SW-dimensional vector $\underline{x}\left(k\right)$ of input variables) and of an output variable y(k) (corresponding to the air temperature at epoch k+1, which has to be forecast starting from $\underline{x}\left(k\right)$).

In a compact notation, one can write:

(9) $$\underline{d}\left(k\right)=\left(\underline{x}\left(k\right),y\left(k\right)\right)$$

where

(10) $$\underline{x}\left(k\right)≜\left(x_{1}\left(k\right),\cdots ,x_{SW}\left(k\right)\right)$$

with

(11) $$x_{i}\left(k\right)=z_{k-i+1}^{\left(T_{\mathrm{samp}}\right)}\;i=1,\ldots ,SW$$

is the vector of the input variable of the model and $y\left(k\right)=z_{k+1}^{\left(T_{\mathrm{samp}}\right)}$ is the true temperature value which has to be estimated by the output of the NN based on $\underline{x}\left(k\right)$. In Figure 7, the generic entry $\underline{d}\left(k\right)$ of $D_{SW}^{\left(T_{\mathrm{samp}}\right)}$, for k>SW, is shown.

As a side note, entries with one or more NaN values—corresponding to missing air temperature values for specific instants of time in the original time series, as explained in Section 4.2—have been discarded and not included in the data sets. Then, each data set is split (randomly) into a training subset and a test subset with a ratio 3:1.

More details on the created data sets, in terms of number of samples in the set (divided among training subset and test subset) and values of SW and $T_{\mathrm{samp}}$, can be found in Table 2.

### 4.5. Models Training

**Figure 8:**
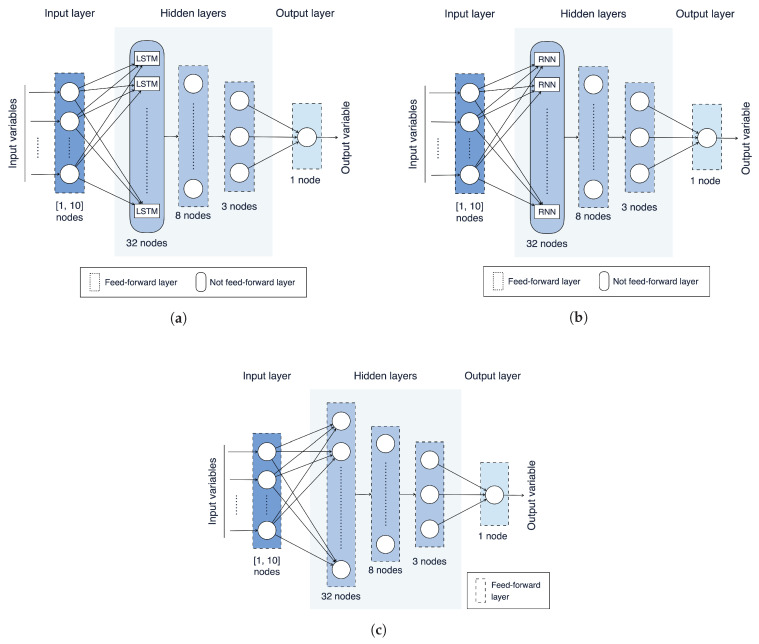
NN models evaluated in this paper: (**a**) LSTM-based; (**b**) RNN-based; (**c**) ANN-based.

The NN models evaluated in this paper, based on LSTMs, RNNs, and ANNs, are shown in Figure 8a, Figure 8b and Figure 8c, respectively. As can be observed, these models share a similar structure, in terms of number of layers and neurons per layer, with the exception of the first hidden layer, which is not feed-forward and is composed of LSTM or RNN cells for the LSTM-based and the RNN-based models, respectively, while it is feed-forward for the ANN-based model. Moreover, as can be expected, the number of neurons in the input layer may vary from 1 to 10, according to the adopted values of SW.

Each model has been trained on the 70 data sets engineered in the previous stages (more precisely, on training subsets obtained from these data sets) using the BP algorithm [23] and considering the RMSE as loss function. From a practical point of view, Python v3.8.6 and the Keras framework v2.4.3 [32] have been used. Moreover, with regard to the other NN’s learning parameters, the following values have been set: learning rate = 0.002, batch size = 20, and number of epochs = 20. In order to perform the fairest possible comparison among these NN models, the values of these parameters have been kept fixed for the three models. In all cases, the number of hidden layers and the number of neurons of the hidden layers are kept fixed as well in all NN models: they are set to 3 and to 32/8/3, respectively.

### 4.6. “Old Model” Re-Training

In order to fairly compare the novel models proposed in this paper with the model presented in [19], the latter (referred in the following as “old model”) has been re-trained with additional new data, namely, meteorological data and sensor data (related to air temperatures) gathered between the beginning of August 2019 and the end of November 2020. As a side note, the additional data (collected in 6 more months with regard to the time interval considered in [19]) allow to increase the data set size of the already deployed model from 5346 to 7919.

## 5. Experimental Results

### 5.1. Sliding Window and Sampling Interval

**Figure 9:**
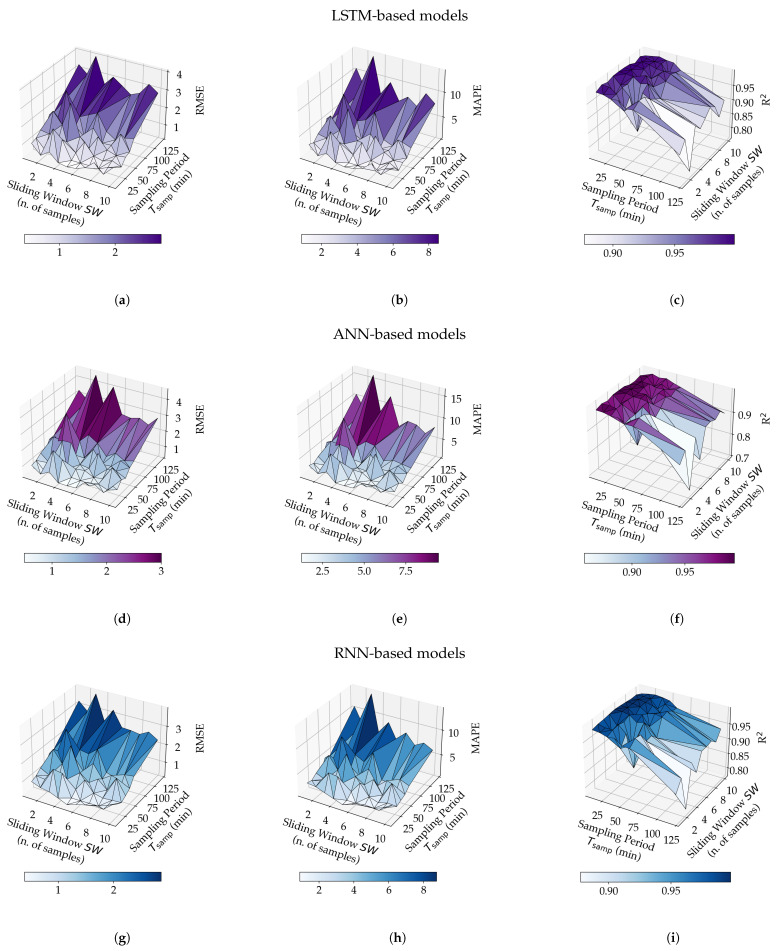
Experimental results with the three proposed NN-based models, namely, LSTM (**a**,**b**,**c**), ANN (**d**,**e**,**f**), and RNN (**g**,**h**,**i**), for different values of SW and $T_{\mathrm{samp}}$, in terms of RMSE (**a**,**d**,**g**), MAPE (**b**,**e**,**h**) and R${}^{2}$ (**c**,**f**,**i**).

In order to evaluate the influence, in terms of prediction performance, of the values of SW and $T_{\mathrm{samp}}$ on the three proposed models (with reference to Figure 8), the RMSE, the MAPE, and R${}^{2}$ have been calculated for each of the 210 models obtained in the training phase (i.e., 3 models for each of the 70 engineered data sets). The values of the three metrics, considering the various models, are shown, with a three-dimensional representation, in Figure 9.

It is noteworthy to remark that the values of RMSE, MAPE, and R${}^{2}$ presented in Figure 9 (as well as those shown in the remainder of the paper) refer to the evaluation of the different models on the test sets (namely, set of data which have not been employed during the models’ training phase). Therefore, the corresponding performance analysis refers directly to the test sets.

Considering the results in Figure 9, the following trends, generally valid for all the evaluated NNs, can be highlighted. *First*, regardless of the value of SW, small values of $T_{\mathrm{samp}}$ are associated with low values of RMSE (namely, a RMSE < 1 ${}^{\circ }C$ for $T_{\mathrm{samp}}=10$ or 20 or 30 min, as shown in Figure 9a,d,g). On the other hand, high values of RMSE and, thus, degraded prediction performance, are obtained for higher values of $T_{\mathrm{samp}}$ (e.g., $T_{\mathrm{samp}}=120$ min). *Second*, the MAPE metric shows the same trend in all cases (as shown in Figure 9b,e,h). *Third*, R${}^{2}$ is higher the smaller $T_{\mathrm{samp}}$ is (i.e., $T_{\mathrm{samp}}=10$ min), but decreases for increasing values of $T_{\mathrm{samp}}$. Moreover, increasing SW leads to an increase of R${}^{2}$ (as shown in Figure 9c,f,i).

**Figure 10:**
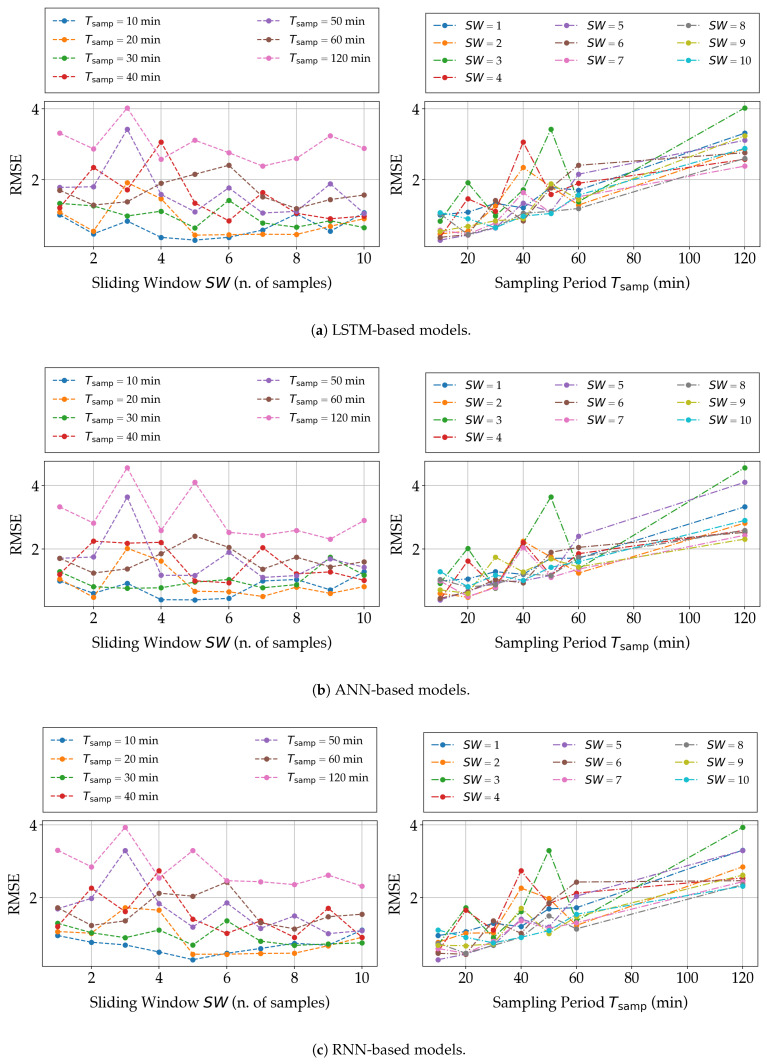
Experimental results, in terms of RMSE, on the three proposed NN-based models, namely, (**a**) LSTM, (**b**) ANN, and (**c**) RNN, for different values of SW and $T_{\mathrm{samp}}$.

The first trend is also confirmed by the bi-dimensional charts shown in Figure 10, in which the prediction performance of the three NN-based models, in terms of RMSE, is shown as a function of either SW (with $T_{\mathrm{samp}}$ as a parameter) or $T_{\mathrm{samp}}$ (with SW as a parameter). In other words, the plots in Figure 10 are obtained by projecting the three-dimensional plots in Figure 9 onto the two vertical planes.

Furthermore, the minimum, maximum, and average values of the three selected evaluation metrics, over the 210 considered models, are listed in Table 3, in detail with the corresponding design parameters (namely, SW and $T_{\mathrm{samp}}$) referring to minimum and maximum values. As can be seen from Figure 9 and Table 3, some of the considered models reach a satisfactory prediction performance on test sets while, on the other hand, some others are less accurate. An example of less accurate model is the ANN-based model trained with ($T_{\mathrm{samp}}$, SW) = (120, 3) which, on the test set, is characterized by RMSE = 4.561 ${}^{\circ }C$, MAPE = 16.35%, and R${}^{2}$ = 0.699.

Less accurate models—namely, those with RMSE >1 ${}^{\circ }C$, MAPE >3%, and R${}^{2}<0.980$ on at least one test set—will not be considered in the following analysis, although their experimental results have been included in Figure 9. In fact, since one of the goals of this paper is to discuss the influence of the dimension of the sliding window and the value of the sampling interval on the model prediction performance exhaustively, even less-performing models have been considered in Figure 9 and discussed in the previous paragraphs.

### 5.2. NN Architecture

In order to fairly compare the prediction performance of the proposed NN architectures (namely, LSTM, RNN, and ANN) over a subset of the 70 data sets generated in the previous phases, 17 models, trained on the same 17 data sets, have been selected for each type of NN architecture. In detail, all the data sets $D_{SW}^{\left(T_{\mathrm{samp}}\right)}$ in correspondence to which the LSTM, RNN, and ANN models trained with $T_{\mathrm{samp}}\in \left\{10,20,30,\ldots ,120\right\}$ and SW∈{1,2,…,10} have RMSE ≤1 ${}^{\circ }C$, MAPE ≤3%, and R${}^{2}\geq 0.980$ on test sets, have been selected. The resulting prediction performance is summarized in Table 4.

**Figure 11:**
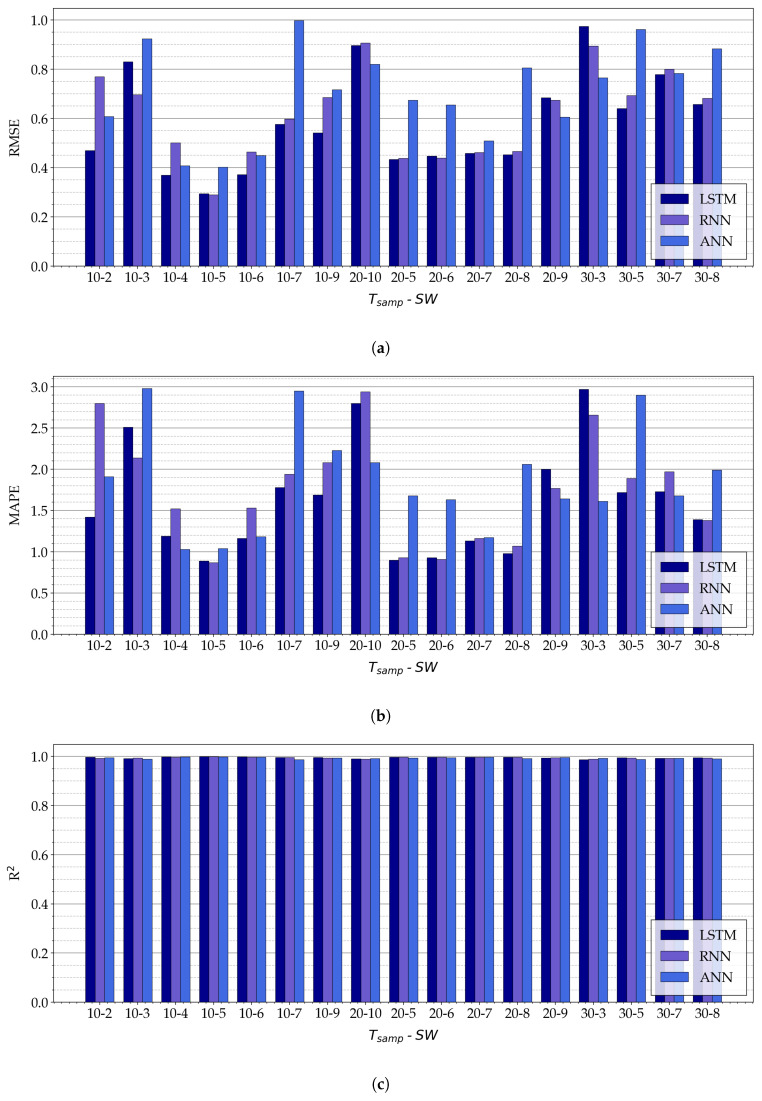
Experimental results on the three proposed NN-based models, namely, LSTM, ANN, and RNN, for a few relevant combination of SW and $T_{\mathrm{samp}}$, expressed in terms of (**a**) RMSE, (**b**) MAPE, and (**c**) R${}^{2}$.

The experimental results (expressed in terms of RMSE, MAPE, and R${}^{2}$) obtained with the three proposed NN-based models for the selected values of SW and $T_{\mathrm{samp}}$, are shown in Figure 11.

As can be seen in Figure 11a,b, for fixed values of SW and $T_{\mathrm{samp}}$, there is not a NN architecture which has the best performance (in terms of RMSE and MAPE) in all cases. For example, the LSTM model has the best performance in terms of RMSE with $T_{\mathrm{samp}}=10$ and SW=4, while for $T_{\mathrm{samp}}=10$ and SW=5 the RNN model is the most accurate.

In general, better results (in terms of the adopted metrics) are given by the following pairs of values of ($T_{\mathrm{samp}}$, SW): (10,4), (10,5), (10,6), (20,5), (20,6), (20,7), and (20,8). For these values, RNN and LSTM models have RMSE ≤ 0.5 ${}^{\circ }C$, MAPE < 1.6%, and R${}^{2}\geq 0.997$.

To conclude, the overall model with the best performance (with regard to the considered metrics) is the RNN model with ($T_{\mathrm{samp}}$, SW) =(10,5), which guarantees a RMSE = 0.289 ${}^{\circ }C$, a MAPE = 0.87%, and a R${}^{2}$ = 0.999. It should be remarked that very similar results have been achieved with the LSTM model with the same values of $T_{\mathrm{samp}}$ and SW (namely, RMSE = 0.294 ${}^{\circ }C$, MAPE =0.98%, and R${}^{2}=0.999$).

### 5.3. Performance Analysis and Literature Comparison

The experimental results of the model presented in [19] (obtained with the data set available in [19]), together with the results of the same model re-trained with a data set containing additional samples (as described in Section 4.5) and with the three models (ANN, RNN, and LSTM) built with the data set $D_{5}^{\left(10\right)}$, are shown in Table 5. As can be seen from Table 5 that the prediction performance of the re-trained model degrades with respect to that in [19]. One possible reason behind this behavior could be that, although re-training involves additional samples (and, therefore, should be, in principle, more accurate), it may happen that the supplementary data are more unbalanced (e.g., in terms of number of collected samples per month, as shown in Figure 4), thus reducing the final accuracy. Moreover, if the model presented in [19] are compared, in terms of prediction performance, with the three models with lowest RMSE obtained with the data set $D_{5}^{\left(10\right)}$ (namely: ${\mathrm{LSTM}}_{5}^{\left(10\right)}$, ${\mathrm{RNN}}_{5}^{\left(10\right)}$, and ${\mathrm{ANN}}_{5}^{\left(10\right)}$), one can conclude that the RMSEs of the latter are slightly lower. Indeed, the RMSE of the model in [19] is higher than the last three collected in Table 5 by at least 1 ${}^{\circ }C$, the MAPE is higher by approximately 3%, and the R${}^{2}$ is lower by approximately 0.24.

In Table 5, the architectural and computational complexities of the considered models are evaluated. More precisely, the architectural and computational complexities can be expressed, respectively, in terms of number of parameters to be used by the model and MAC operations (see Section 2). Analyzing the results in Table 5, the LSTM-based model (namely, ${\mathrm{LSTM}}_{5}^{\left(10\right)}$) is the most architecturally and computationally complex (with respect to the other NNs). The RNN-based model (namely, ${\mathrm{RNN}}_{5}^{\left(10\right)}$) ranks second and is then followed by the model presented in [19] and its re-trained version. The ${\mathrm{ANN}}_{5}^{\left(10\right)}$ model is the “lightest” model, with only 464 parameters and 464 MAC operations. In other words, ${\mathrm{ANN}}_{5}^{\left(10\right)}$ is the one with lowest architectural and computational complexities.

Finally, the NetScore metric (introduced in Section 2) has been calculated for the 5 models detailed in Table 5, with the goal to identify which model reaches the best trade-off between prediction performance and complexity. With reference to Equation (5), one of the parameters required to calculate the NetScore (for a target NN model N) is the model’s accuracy a(N). This accuracy is an evaluation metric generally adopted in classification tasks, which can be defined, over a target data set, as the percentage of samples correctly attributed to their classes by a predictor. Since the air temperature forecasting is practically a regression task and, for this reason, the canonical definition of the accuracy cannot be applied for the evaluation of the models, the authors re-define the concept of accuracy—and, thus, the meaning of a(N) in Equation (5)—in order to calculate the NetScore metric for the models. In detail, the authors define a threshold parameter T and an indicator function I which may assume, for each sample $\underline{d}\left(k\right)=\left(\underline{x}\left(k\right),y\left(k\right)\right)$ in the test subset of the model to be evaluated, a binary value as follows:

(12) $$\begin{array}{ccc} I\left(\underline{d}\left(k\right)\right)=I\left(y\left(k\right),\hat{y}\left(k\right)\right)=U(T-|y\left(k\right)-\hat{y}\left(k\right)\left|\right) & = & \left\{\begin{array}{cc} 1 & \mathrm{if}\;|y\left(k\right)-\hat{y}\left(k\right)|<T\\ 0 & \mathrm{otherwise} \end{array}\right. \end{array}$$

where: U(·) is the unit step function; |·| is the modulo operator; $\underline{d}\left(k\right)$ is the *k*-th entry in the test subset; $\underline{x}\left(k\right)$ is the vector of the input samples of the test subset entry $\underline{d}\left(k\right)$; y(k) and $\hat{y}\left(k\right)$ are the actual (in the entry $\underline{d}\left(k\right)$) and the forecast by the NN (with input $\underline{x}\left(k\right)$) values of air temperature for the *k*-th test subset entry, respectively; and T is a threshold value.

Therefore, the accuracy a(N) for the model N over all the samples in the test subset is defined as follows:

(13) $$\begin{array}{ccc} a(N) & = & \frac{100}{N_{\mathrm{tst}}}\sum_{r=1}^{N_{\mathrm{tst}}}I\left(y\left(k\right),\hat{y}\left(k\right)\right), \end{array}$$

where $N_{\mathrm{tst}}$ is the number of samples in the test subset; y(k) and $\hat{y}\left(k\right)$ are the actual and the forecast values (of air temperature) for the *k*-th test subset entry, respectively.

The value of T is set to 1 ${}^{\circ }C$: from a conceptual point of view, this means that a future air temperature value $\hat{y}\left(k\right)$ forecast by the model is considered as correct (e.g., $I(y\left(k\right),\hat{y}\left(k\right))=1$) if the absolute value of the difference between actual and forecast air temperature values ($|y\left(k\right)-\hat{y}\left(k\right)|$) is lower than 1 ${}^{\circ }C$. The exponential coefficients of the NetScore metric are set to their default values, namely α=2 and β=γ=0.5. The corresponding NetScore values are listed in Table 5.

As can be seen from the results in Table 5, the highest NetScore is reached by the ${\mathrm{ANN}}_{5}^{\left(10\right)}$ model, followed by the ${\mathrm{RNN}}_{5}^{\left(10\right)}$ model, and then by the model the authors presented in [19]. On the other hand, the re-trained version of [19] and the ${\mathrm{LSTM}}_{5}^{\left(10\right)}$ model return the lowest NetScore, thus highlighting that the trade-off between prediction performance and complexity achieved by these models is rather unbalanced. Indeed, the prediction performance of the re-trained version of [19] is lower than that of the ${\mathrm{LSTM}}_{5}^{\left(10\right)}$ model (e.g., with RMSE equal to 2.28 ${}^{\circ }C$ and 0.294 ${}^{\circ }C$, respectively). On the other hand, computational and architectural complexities of the re-trained version of [19] are significantly lower than those of the ${\mathrm{LSTM}}_{5}^{\left(10\right)}$ model, with a ratio between the number of MAC operations of the re-trained model from [19] and ${\mathrm{LSTM}}_{5}^{\left(10\right)}$ approximately equal to 1:22. Overall, the ${\mathrm{ANN}}_{5}^{\left(10\right)}$ is the model to be preferred for execution on edge devices.

With regard to the reference works listed in Table 1, it can be noted that the prediction performance of the three best models proposed in this paper (namely, ${\mathrm{LSTM}}_{5}^{\left(10\right)}$, ${\mathrm{RNN}}_{5}^{\left(10\right)}$, and ${\mathrm{RNN}}_{5}^{\left(10\right)}$) are comparable with those provided in these works. In particular:

- the proposed NN-based models have a RMSE in the range 0.289÷0.402 ${}^{\circ }C$, lower than that in [3,14,27,28] and slightly higher than that in [15,16];
- the considered NN-based models have a MAPE in the range 0.87÷1.04%, thus lower than that in [15,27];
- the value of R${}^{2}$ of the considered NN-based models is higher than those of all the references listed in Table 1.

Finally, as detailed in Section 2, the literature works listed in Table 1 do not provide details on the complexity of their deployed models: this prevents a direct comparison, from a complexity-performance trade-off perspective, between them and the NN-based models proposed in this paper.

### 5.4. Possible Application Scenario and Reference Architecture

**Figure 12:**
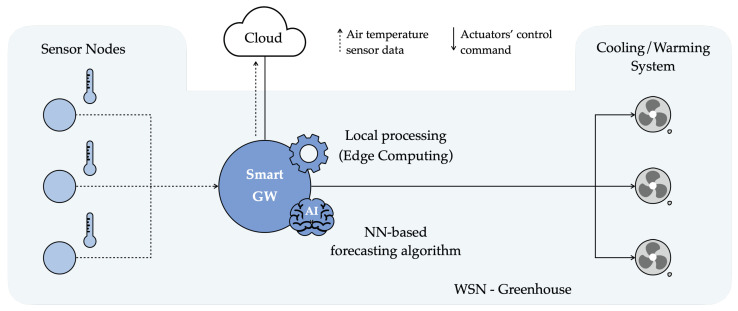
Possible reference architecture and application scenario for the developed forecasting model.

From an implementation point of view, the NN model selected in Section 5.3 (namely, ${\mathrm{ANN}}_{5}^{\left(10\right)}$) can be deployed on a real IoT edge device, denoted as Smart GW, such as a Raspberry Pi (RPi) [33], which represents a popular Single Board Computer (SBC) in EdgeAI applications [34,35]. Targeting a Smart Farming scenario, the Smart GW can be placed inside a greenhouse, which hosts also a WSN deployed to monitor the internal air temperature, and acts as a data collector for the WSN’s SNs, as shown in Figure 12. More precisely, SNs in the greenhouse forward-sensed air temperature data to the Smart GW thanks to a wireless connection existing (or introduced specifically for this purpose) in the greenhouse, e.g., a Wi-Fi network. Then, the information arriving from the SNs is temporarily stored by the GW in its internal memory and, if necessary, also forwarded to the Cloud (if an Internet access point is available around the greenhouse).

In a demonstration greenhouse in the AFarCloud project [30], the Smart GW is also connected with the cooling/warming system installed inside the greenhouse and can control it to regulate the internal air temperature and humidity levels. If needed, as well as when the proper number of consecutive air temperature values have been collected from SNs—in turn, to be used as inputs for the forecasting model (e.g., 5 values for ${\mathrm{ANN}}_{5}^{\left(10\right)}$)—the Smart GW runs the forecasting algorithm, obtaining a future predicted air temperature value. According to the forecast value, the Smart GW decides if the cooling/warming system needs to be activated, deactivated, or maintained in its current operational state. As an example, if the predicted air temperature value is higher than a certain threshold which cannot be exceeded inside the greenhouse, the GW can send a command to the internal cooling system requiring an opening actuation, thus avoiding the internal air temperature from reaching the unwanted (and dangerous) forecast value.

To conclude, the adoption of EdgeAI solutions has an extremely positive impact on greenhouse management, which, as previously mentioned, needs to tightly control the internal microclimate. Indeed, being able to predict the trend of internal environmental variables (e.g., air temperature), such an IoT/EdgeAI system allows to preemptively schedule actions and, thus, to more effectively pilot greenhouse’s actuators to maintain (within the greenhouse) the best-growing conditions for the internal cultivation. Moreover, executing AI algorithms and taking decisions at the edge, rather than in the Cloud, makes greenhouse management more robust (against connectivity problems) and reduces latency. As a last comment, the proposed IoT-oriented EdgeAI NN-based forecasting can be effectively integrated into the LoRaFarM platform [8] simply as a new additional software module.

## 6. Conclusions

In this paper, the authors have presented an NN-based approach, based on time series, expedient to design a forecasting algorithm to be embedded into a Smart GW, in turn acting as a data collector for SNs measuring air temperature inside a greenhouse. In comparison to the approach in [19], based on an ANN-based prediction model to predict temperature values considering weather conditions external to the greenhouse as input sources, in this paper the authors have considered indoor air temperature values, collected inside the greenhouse, as NN models’ input variables. In detail, three types of NN architectures—namely, LSTM, RNN, and ANN—have been investigated to determine the one with the best performance and the lowest complexity (from both computational and architectural viewpoints). This is fundamental to run a NN model on an edge device with constrained capabilities—namely, the Smart GW presented and discussed in [19].

According to the obtained experimental results, it can be concluded that the three proposed models (namely, LSTM, RNN, and ANN) reach a prediction performance comparable to that of literature works improve over that of the model proposed in [19]. In particular, the three best performing algorithms, obtained with a sampling interval $T_{\mathrm{samp}}=10$ min and using SW=5 input variables, reach a RMSE in the range of 0.289÷0.402 ${}^{\circ }C$, a MAPE in the range of 0.87÷1.04%, and a R${}^{2}$≥0.997. Moreover, the results show that the ANN-based model has lower computational complexity, in terms of the number of MAC operations and parameters to store, than the one proposed in [19]. Overall, ${\mathrm{ANN}}_{5}^{\left(10\right)}$ is the NN model guaranteeing the best performance-complexity trade-off. In general, the design and implementation of NN-based accurate prediction algorithms, yet with a computational complexity compatible with the processing resources of IoT edge devices, is an interesting and promising research topic, not extensively discussed in recent literature. This is even more true for the greenhouse’s internal variables forecasting, which this paper has tried to address in the more comprehensive possible way.

As a final remark, future research directions may involve the performance evaluation of the proposed NN-based algorithms on different types of IoT edge devices, based on relevant metrics for the algorithms’ online execution. To this end, illustrative interesting performance metrics are the following: (i) the time required by the device to run the algorithm in a real scenario; (ii) the (flash) memory space the device needs to store the model, as well as the RAM memory required to run it; and (iii) the IoT edge device’s power consumption when running the NN-based algorithms to forecast a future air temperature value. Another possible direction includes the application of the same methodology followed in this paper (in order to build an air temperature forecasting model) to develop algorithms able to predict other types of inner variables (rather than air temperature) which, in turn, are relevant for greenhouse cultivation and, thus, have to be monitored and controlled (e.g., air humidity).

## Figures and Tables

**Table 1:** Representative literature papers in the context of air temperature forecasting inside greenhouses through the usage of NNs.

Ref.	NN Model			Performances (on Test Set)			Data Set Details		
	Input Variables	Architectural Type	Training Algorithm	RMSE (${}^{\circ }$C)	MAPE (%)	R${}^{2}$	Size (Samples No)	Collection Interval	Sampling Interval
[3]	External temperature and solar radiation, wind speed, heater temperature, datetime reference	ANN	BP, CGA	2.5–3.0	N/A	N/A	1368	≈2 months	1 h
[14]	Internal solar radiation, air temperature and humidity, and soil moisture, CO${}_{2}$, atmospheric pressure, datetime reference	ANN	BP	0.839	N/A	0.977	≈87,408	19 months	10 min
[15]	External solar radiation and temperature, wind speed	ANN, RBF	BP	0.20±0.02, 0.13±0.01	0.93±0.10, 0.59±0.07	0.76±0.05, 0.89±0.03	N/A	N/A	N/A
[16]	External solar radiation, heater temperature, internal air temperature and humidity, wind speed, history of actuators, shadow screen	RBF	BP, LM	0.0019	N/A	N/A	1728	12 days	10 min
[19]	External apparent temperature, dew point, air humidity, air temperature and UV index, datetime reference	ANN	BP	1.50	4.91	0.965	5346	10 months	1 h
[28]	External temperature, solar radiation and humidity, wind speed and direction, history of actuators	ANN, RNN-LSTM, NARX	BP	0.89–0.94, 0.45–0.71, 0.52–1.32	N/A	0.94, 0.96–0.97, 0.86–0.96	≈470,000	1 year	5, 10, 15, 20, 25, 30 min
[27]	Internal air and soil temperature, internal solar radiation, humidity and CO${}_{2}$	RNN	BP	0.865	1.7	0.925	1152	8 days	10 min

**Table 2:** Details concerning the engineered data sets, in terms of number of samples, SW and $T_{\mathrm{samp}}$.

Data Set	$T_{\mathrm{samp}}$ [min]	SW [Samples]	Size [Samples]	Training Subset Size [Samples]	Test Subset Size [Samples]	Data Set	$T_{\mathrm{samp}}$ [min]	SW [Samples]	Size [Samples]	Training Subset Size [Samples]	Test Subset Size [Samples]
$D_{1}^{\left(10\right)}$	10	1	36,330	27,248	9082	$D_{10}^{\left(10\right)}$	10	10	32,696	24,522	8174
$D_{2}^{\left(10\right)}$	10	2	35,828	26,871	8957	$D_{3}^{\left(10\right)}$	10	3	35,363	26,523	8840
$D_{4}^{\left(10\right)}$	10	4	34,923	26,193	8730	$D_{5}^{\left(10\right)}$	10	5	34,512	25,884	8628
$D_{6}^{\left(10\right)}$	10	6	34,118	25,589	8529	$D_{7}^{\left(10\right)}$	10	7	33,734	25,301	8433
$D_{8}^{\left(10\right)}$	10	8	33,366	25,025	8341	$D_{9}^{\left(10\right)}$	10	9	33,020	24,765	8255
$D_{1}^{\left(120\right)}$	120	1	2985	2239	746	$D_{10}^{\left(120\right)}$	120	10	2457	1843	614
$D_{2}^{\left(120\right)}$	120	2	2912	2184	728	$D_{3}^{\left(120\right)}$	120	3	2843	2133	710
$D_{4}^{\left(120\right)}$	120	4	2781	2086	695	$D_{5}^{\left(120\right)}$	120	5	2723	2043	680
$D_{6}^{\left(120\right)}$	120	6	2666	2000	666	$D_{7}^{\left(120\right)}$	120	7	2611	1959	652
$D_{8}^{\left(120\right)}$	120	8	2558	1919	639	$D_{9}^{\left(120\right)}$	120	9	2507	1881	626
$D_{1}^{\left(20\right)}$	20	1	18,102	13,577	4525	$D_{10}^{\left(20\right)}$	20	10	16,006	12,005	4001
$D_{2}^{\left(20\right)}$	20	2	17,812	13,359	4453	$D_{3}^{\left(20\right)}$	20	3	17,539	13,155	4384
$D_{4}^{\left(20\right)}$	20	4	17,286	12,965	4321	$D_{5}^{\left(20\right)}$	20	5	17,050	12,788	4262
$D_{6}^{\left(20\right)}$	20	6	16,822	12,617	4205	$D_{7}^{\left(20\right)}$	20	7	16,605	12,454	4151
$D_{8}^{\left(20\right)}$	20	8	16,399	12,300	4099	$D_{9}^{\left(20\right)}$	20	9	16,196	12,147	4049
$D_{1}^{\left(30\right)}$	30	1	12,067	9051	3016	$D_{10}^{\left(30\right)}$	30	10	10,618	7964	2654
$D_{2}^{\left(30\right)}$	30	2	11,862	8897	2965	$D_{3}^{\left(30\right)}$	30	3	11,678	8759	2919
$D_{4}^{\left(30\right)}$	30	4	11,504	8628	2876	$D_{5}^{\left(30\right)}$	30	5	11,341	8506	2835
$D_{6}^{\left(30\right)}$	30	6	11184	8388	2796	$D_{7}^{\left(30\right)}$	30	7	11,030	8273	2757
$D_{8}^{\left(30\right)}$	30	8	10,885	8164	2721	$D_{9}^{\left(30\right)}$	30	9	10,749	8062	2687
$D_{1}^{\left(40\right)}$	40	1	9008	6756	2252	$D_{10}^{\left(40\right)}$	40	10	7690	5768	1922
$D_{2}^{\left(40\right)}$	40	2	8829	6622	2207	$D_{3}^{\left(40\right)}$	40	3	8656	6492	2164
$D_{4}^{\left(40\right)}$	40	4	8495	6372	2123	$D_{5}^{\left(40\right)}$	40	5	8341	6256	2085
$D_{6}^{\left(40\right)}$	40	6	8196	6147	2049	$D_{7}^{\left(40\right)}$	40	7	8062	6047	2015
$D_{8}^{\left(40\right)}$	40	8	7931	5949	1982	$D_{9}^{\left(40\right)}$	40	9	7806	5855	1951
$D_{1}^{\left(50\right)}$	50	1	7220	5415	1805	$D_{10}^{\left(50\right)}$	50	10	6185	4639	1546
$D_{2}^{\left(50\right)}$	50	2	7079	5310	1769	$D_{3}^{\left(50\right)}$	50	3	6944	5208	1736
$D_{4}^{\left(50\right)}$	50	4	6816	5112	1704	$D_{5}^{\left(50\right)}$	50	5	6695	5022	1673
$D_{6}^{\left(50\right)}$	50	6	6584	4938	1646	$D_{7}^{\left(50\right)}$	50	7	6479	4860	1619
$D_{8}^{\left(50\right)}$	50	8	6376	4782	1594	$D_{9}^{\left(50\right)}$	50	9	6280	4710	1570
$D_{1}^{\left(60\right)}$	60	1	6006	4505	1501	$D_{10}^{\left(60\right)}$	60	10	5146	3860	1286
$D_{2}^{\left(60\right)}$	60	2	5886	4415	1471	$D_{3}^{\left(60\right)}$	60	3	5772	4329	1443
$D_{4}^{\left(60\right)}$	60	4	5668	4251	1417	$D_{5}^{\left(60\right)}$	60	5	5570	4178	1392
$D_{6}^{\left(60\right)}$	60	6	5478	4109	1369	$D_{7}^{\left(60\right)}$	60	7	5389	4042	1347
$D_{8}^{\left(60\right)}$	60	8	5306	3980	1326	$D_{9}^{\left(60\right)}$	60	9	5224	3918	1306

**Table 3:** Minimum (min), maximum (max), and average (avg) values of RMSE, MAPE, and R${}^{2}$ obtained over the 210 trained models.

NN Arch. Type		RMSE [${}^{\circ }$C]			MAPE [%]			R${}^{2}$		
		Value	$T_{\mathrm{samp}}$	SW	Value	$T_{\mathrm{samp}}$	SW	Value	$T_{\mathrm{samp}}$	SW
**ANN**	**Min**	0.402	10	5	1.03	10	4	0.699	120	3
	**Max**	4.561	120	3	16.35	120	3	0.998	10	4, 5
	**Avg**	1.52	N/A	N/A	4.29	N/A	N/A	0.96	N/A	N/A
**RNN**	**Min**	0.290	10	5	0.87	10	5	0.776	120	3
	**Max**	3.933	120	3	14.14	120	3	0.999	10	5
	**Avg**	1.45	N/A	N/A	4.10	N/A	N/A	0.96	N/A	N/A
**LSTM**	**Min**	0.294	10	5	0.89	10	5	0.766	120	3
	**Max**	4.024	120	3	14.08	120	3	0.999	10	5
	**Avg**	1.46	N/A	N/A	4.17	N/A	N/A	0.96	N/A	N/A

**Table 4:** Prediction performances of the three proposed models on a reduced selection of SW and $T_{\mathrm{samp}}$ (those which are better performing).

Data Set	$T_{}$	SW	RMSE [${}^{\circ }$C]			MAPE [%]			R${}^{2}$		
			LSTM	RNN	ANN	LSTM	RNN	ANN	LSTM	RNN	ANN
$D_{2}^{\left(10\right)}$	10	2	0.470	0.769	0.608	1.42	2.80	1.91	0.997	0.992	0.995
$D_{3}^{\left(10\right)}$	10	3	0.830	0.696	0.923	2.51	2.14	2.98	0.991	0.994	0.989
$D_{4}^{\left(10\right)}$	10	4	0.370	0.501	0.407	1.19	1.52	1.03	0.998	0.997	0.998
$D_{5}^{\left(10\right)}$	10	5	0.294	0.289	0.402	0.89	0.87	1.04	0.999	0.999	0.998
$D_{6}^{\left(10\right)}$	10	6	0.371	0.464	0.449	1.16	1.53	1.18	0.998	0.997	0.997
$D_{7}^{\left(10\right)}$	10	7	0.577	0.598	0.997	1.78	1.94	2.95	0.996	0.996	0.987
$D_{9}^{\left(10\right)}$	10	9	0.542	0.685	0.717	1.69	2.08	2.23	0.996	0.994	0.993
$D_{5}^{\left(20\right)}$	20	5	0.434	0.438	0.674	0.90	0.93	1.68	0.998	0.998	0.994
$D_{6}^{\left(20\right)}$	20	6	0.447	0.439	0.655	0.93	0.91	1.63	0.997	0.998	0.995
$D_{7}^{\left(20\right)}$	20	7	0.458	0.461	0.509	1.13	1.16	1.17	0.997	0.997	0.997
$D_{8}^{\left(20\right)}$	20	8	0.453	0.466	0.805	0.98	1.07	2.06	0.997	0.997	0.992
$D_{9}^{\left(20\right)}$	20	9	0.684	0.674	0.606	2.00	1.77	1.64	0.994	0.994	0.995
$D_{10}^{\left(20\right)}$	20	10	0.897	0.907	0.820	2.80	2.94	2.08	0.990	0.990	0.992
$D_{3}^{\left(30\right)}$	30	3	0.974	0.894	0.765	2.97	2.66	1.61	0.987	0.989	0.992
$D_{5}^{\left(30\right)}$	30	5	0.640	0.693	0.961	1.72	1.89	2.90	0.995	0.994	0.988
$D_{7}^{\left(30\right)}$	30	7	0.778	0.799	0.782	1.73	1.97	1.68	0.993	0.992	0.992
$D_{8}^{\left(30\right)}$	30	8	0.657	0.682	0.883	1.39	1.38	1.99	0.995	0.994	0.990

**Table 5:** Prediction performance and complexity of a subset of evaluated models, in terms of RMSE, MAPE, R${}^{2}$, accuracy (namely, number of samples predicted with RMSE lower than 1 ${}^{\circ }C$), MAC operations, number of parameters, and NetScore of the models.

Model	RMSE [${}^{\circ }$C]	MAPE [%]	R${}^{2}$	Accuracy [%]	MAC Operations	Parameters Number	NetScore
Model in [19]	1.50	4.91	0.965	48.87	1018	1018	17.05
Re-trained [19]	2.28	6.54	0.931	34.91	1018	1018	3.60
${\mathrm{LSTM}}_{5}^{\left(10\right)}$	0.294	0.89	0.999	99.18	22,192	4625	−0.59
${\mathrm{RNN}}_{5}^{\left(10\right)}$	0.289	0.87	0.999	99.28	5712	1361	25.25
${\mathrm{ANN}}_{5}^{\left(10\right)}$	0.402	1.04	0.998	97.28	464	464	60.31

## Data Availability

Not applicable.

## Abbreviations

The following abbreviations are used in this manuscript:

AFarCloud	Aggregate Farming in the Cloud
AI	Artificial Intelligence
ANN	Artificial Neural Network
BP	Back Propagation
CGA	Conjugate Gradient Algorithm
DL	Deep Learning
DNN	Deep Neural Network
FaaS	Farm-as-a-Service
GW	Gateway
ICT	Information and Communication Technology
IoT	Internet of Things
LM	Levenberg-Marquardt
LSTM	Long Short-Term Memory
MAC	Multiply–ACcumulate
MAPE	Mean Absolute Percentage Error
ML	Machine Learning
MLP	Multi-Layer Perceptron
NARX	Nonlinear AutoRegressive with eXternal input
NN	Neural Network
PSO	Particle Swarm Optimization
R${}^{2}$	Coefficient of determination
RBF	Radial Basis Function
ReLU	Rectified Linear Unit
RMSE	Root Mean Squared Error
RNN	Recurrent Neural Network
SA	Smart Agriculture
SBC	Single Board Computer
SF	Smart Farming
SN	Sensor Node
UI	User Interface
WSN	Wireless Sensor Network
